# Plant_SNP_TATA_Z-Tester: A Web Service That Unequivocally Estimates the Impact of Proximal Promoter Mutations on Plant Gene Expression

**DOI:** 10.3390/ijms23158684

**Published:** 2022-08-04

**Authors:** Dmitry Rasskazov, Irina Chadaeva, Ekaterina Sharypova, Karina Zolotareva, Bato Khandaev, Petr Ponomarenko, Nikolay Podkolodnyy, Natalya Tverdokhleb, Oleg Vishnevsky, Anton Bogomolov, Olga Podkolodnaya, Ludmila Savinkova, Elena Zemlyanskaya, Vadimir Golubyatnikov, Nikolay Kolchanov, Mikhail Ponomarenko

**Affiliations:** 1 Institute of Cytology and Genetics, 630090 Novosibirsk, Russia; 2 Institute of Computational Mathematics and Mathematical Geophysics, 630090 Novosibirsk, Russia; 3 Sobolev Institute of Mathematics, 630090 Novosibirsk, Russia

**Keywords:** TATA-binding protein, TATA box, promoter, gene, expression, plant, development, environmental exposure, mutation, prediction, Web service, verification, correlation, plant hybrid, marker-assisted breeding

## Abstract

Synthetic targeted optimization of plant promoters is becoming a part of progress in mainstream postgenomic agriculture along with hybridization of cultivated plants with wild congeners, as well as marker-assisted breeding. Therefore, here, for the first time, we compiled all the experimental data—on mutational effects in plant proximal promoters on gene expression—that we could find in PubMed. Some of these datasets cast doubt on both the existence and the uniqueness of the sought solution, which could unequivocally estimate effects of proximal promoter mutation on gene expression when plants are grown under various environmental conditions during their development. This means that the inverse problem under study is ill-posed. Furthermore, we found experimental data on in vitro interchangeability of plant and human TATA-binding proteins allowing the application of Tikhonov’s regularization, making this problem well-posed. Within these frameworks, we created our Web service Plant_SNP_TATA_Z-tester and then determined the limits of its applicability using those data that cast doubt on both the existence and the uniqueness of the sought solution. We confirmed that the effects (of proximal promoter mutations on gene expression) predicted by Plant_SNP_TATA_Z-tester correlate statistically significantly with all the experimental data under study. Lastly, we exemplified an application of Plant_SNP_TATA_Z-tester to agriculturally valuable mutations in plant promoters.

## 1. Introduction

The growth of the production of food, medicines, and livestock feed from plants with inexorable growth of population inevitably requires a “quantum leap” [1] in targeted breeding of agricultural plants by means of genomic big data [2]. Synthetic pinpoint optimization of plant gene promoters [3] to adapt plants to various environmental conditions during plant development (e.g., drought under climate change [4]) is becoming a part of the mainstream postgenomic agriculture progress [5] along with the design of hybrids of cultivated plants with their wild congeners [6] and both quantitative trait locus (QTL)- and single-nucleotide polymorphism (SNP) marker-assisted breeding [7]. Recently, a *Faecalibaculum rodentium* Cas9 protein for genome-editing CRISPR/Cas9 systems was found whose protospacer-adjacent motif (PAM) “NNTA” matches TATA-binding protein (TBP)-binding sites of eukaryotic promoters [8]. The ability of this protein to directly target the TATA box was confirmed for TATA-containing promoters of human genes *ABCA1*, *UCP1,* and *RANKL* [8].

Of note, the TATA box is the most conserved regulatory site in terms of its nucleotide sequence. Moreover, it is the only mandatory element in a multitude of TATA-containing eukaryotic promoters [9,10,11,12]. Moreover, TBP-binding sites, whose canonical form is the TATA box, are obligatory for primary transcription initiation [13,14]; specifically, a stronger TBP binding affinity for a promoter of a given gene indicates a higher expression level of this gene [15]. That is why, within 90 bp, proximal promoter mutations [16,17] that alter the abovementioned TBP–promoter affinity during TBP sliding along the promoter DNA helix in order to search for proper TBP-binding sites [18] can affect expression levels of the corresponding genes.

The structure and function of plant promoters have been exhaustively described previously [19]. For instance, tcacTATATATAg represents the consensus sequence for TATA boxes in plants [20]. Plant promoters are TA/CG-deficient and TG/CT-rich [21], and their 500 bp region in front of their transcription start sites (TSSes) is enriched with *cis*-regulatory elements and contains few SNPs [22]. However, experimental verification of effects of plant promoter mutations on gene expression is labor-, cost-, and time-consuming. Therefore, a bioinformatic toolbox capable of estimating the effects of such mutations may facilitate agricultural progress [23], provide new insights into the transcriptional regulation of plant development [24] and response to changing environment [25], and prevent negative effects of exogenous plant macromolecules on both the health and the microbiota of humans via food [26].

In our previous studies, we created a Web service Human_SNP_TATA_Z-tester (http://wwwmgs.bionet.nsc.ru/cgi-bin/mgs/tatascan_fox/start.pl, accessed on 10 June 2022) for estimating the effects of SNPs within 90 bp proximal promoters of human genes on disease development [27,28]. It uses a step-by-step approximation [29]: (i) TBP slides along DNA [18]; (ii) TBP stops at the best TBP-binding site [30,31]; (iii) the TBP–promoter complex is fixed by the DNA bending at a right angle [32], as proven experimentally [33]. Subsequently, using Human_SNP_TATA_Z-tester, we analyzed 15243 SNPs, which yielded 3229 candidate SNP markers aggravating or relieving the development of human disorders, such as subfertility [28,34], obesity [35], hypertension [36], cognitive disorders [37], atherosclerosis [38], Alzheimer’s disease [39,40], hematopoietic disorders [41], and many others (for a review, see [42]). Lastly, from article to article, we selectively experimentally verified these predictions, as did some independent researchers (e.g., see [43]). 

In the present work, we expanded both plant and mutation areas of our research to create a Web service, Plant_SNP_TATA_Z-tester, which allows estimating the effects of mutations in plant promoters on gene expression (http://wwwmgs.bionet.nsc.ru/cgi-bin/mgs/tatascan_plant/start.pl, accessed on 10 June 2022). We verified its results using all experimental data that we could find in the PubMed database [44], as stored in our knowledge base Plant_SNP_TATAdb (https://www.sysbio.ru/Plant_SNP_TATAdb/, accessed on 10 June 2022) created in this work. Lastly, we discuss how to use Plant_SNP_TATA_Z-tester for assessing the effects of agriculturally valuable mutations in plant promoters on gene expression during wheat development, namely, wheat winter and spring lines, as well as their hybrids with wild congeners.

## 2. Results

### 2.1. The Experimental Data—On the Effects of Mutations in Plant Promoters on Gene Expression—That We Could Find in the PubMed Database in Order to Investigate Them in This Work

Using the PubMed database [44], we collected all available experimental datasets reflecting the effects of mutations in plant proximal promoters on gene expression [45,46,47,48,49,50,51,52,53,54,55,56,57,58,59] (Table 1). 

A total of 242 variants of plant promoters were quantitatively characterized in terms of their effects on gene expression under experimental conditions in vitro, ex vivo, and in vivo (Table 1: datasets 1–4, 5–10, and 11–12, respectively). Each experimental dataset contained at least five variants of promoters necessary for their adequate statistical analysis. First of all, two datasets (1 and 2) reflected the functioning of the plant TBP-1 from thale cress (*Arabidopsis thaliana*, dataset 2: atTBP) compared to the human TBP as a reference (dataset 1: hsTBP) [53]. This is a well-known phenomenon of in vitro interchangeability of plant and human TBPs [60]. Furthermore, there are datasets containing information about plant TBPs from rice (*Oryza sativa*, dataset 3) and tobacco (*Nicotiana plumbaginifolia*; datasets 4–12). Transcription was performed by means of RNA polymerase II (datasets 1, 2, 7, 8, 10, 11, and 12) or III (datasets 3–6 and 9). Lastly, there were mutations in both natural promoters (datasets 1–8) and prototypical artificial promoters (datasets 9–12).

### 2.2. The Ill-Posed Inverse Problem under Study and Its Solution via Tikhonov’s Regularization

**Figure 1:**
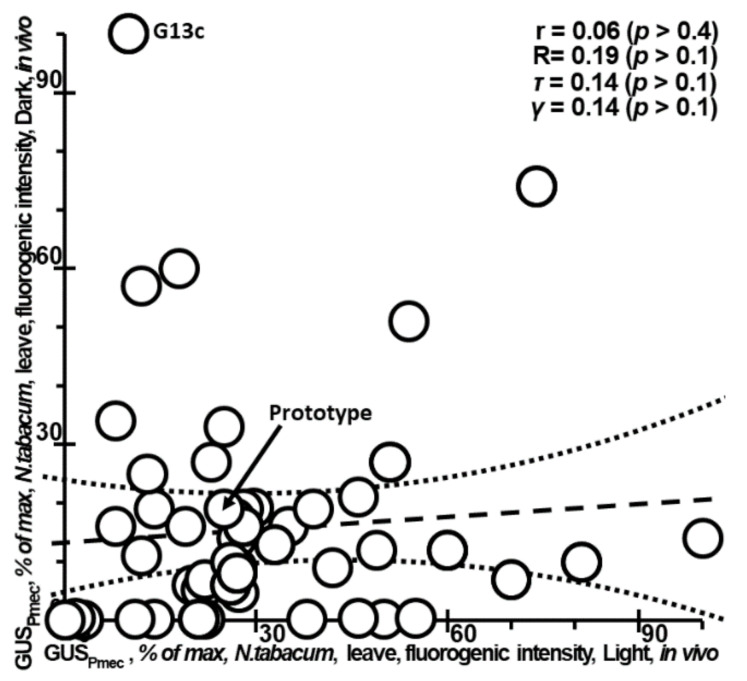
The effects of mutation within the analyzed artificial *Pmec* promoter on the β-glucuronidase (GUS) activity of tobacco under the experimental conditions “light” (*x*-axis) and “dark” (*y*-axis) in vivo [53] do not correlate with each other, thereby casting doubt on both the existence and the uniqueness of the uniform estimate for the mutational effects of plant proximal promoters on gene expression under various environmental conditions, usually called an ill-posed inverse problem [61]. *Legend*: circle, the prototype (arrow, →), or a mutant variant of the studied artificial promoter *Pmec* for plants; dashed and dotted lines are linear regression and limits of its 95% confidence interval, as calculated in the Statistica software (Statsoft^TM^, Tulsa, OK, USA); r, R, τ, γ, and *p* are the linear correlation, Spearman’s rank correlation, Kendall’s rank correlation, Goodman–Kruskal generalized correlation coefficients, and their statistical significance, respectively.

It is noteworthy that we found no correlation in the effects of the same mutations in the same plant promoter on the expression of the same gene between tobacco plants grown in the dark or under light (datasets 11 and 12 in Table 1; Figure 1) [53]. This finding casts doubt on both the existence and the uniqueness of the solution that describes the transcriptional outcome of mutations in plant proximal promoters under various environmental and developmental conditions. This means that, in different specimens of the same plant grown under different environmental conditions during development, the inverse problem about how a given mutation within a given proximal promoter affects gene expression seems to be quite ill-posed [61]. 

**Figure 2:**
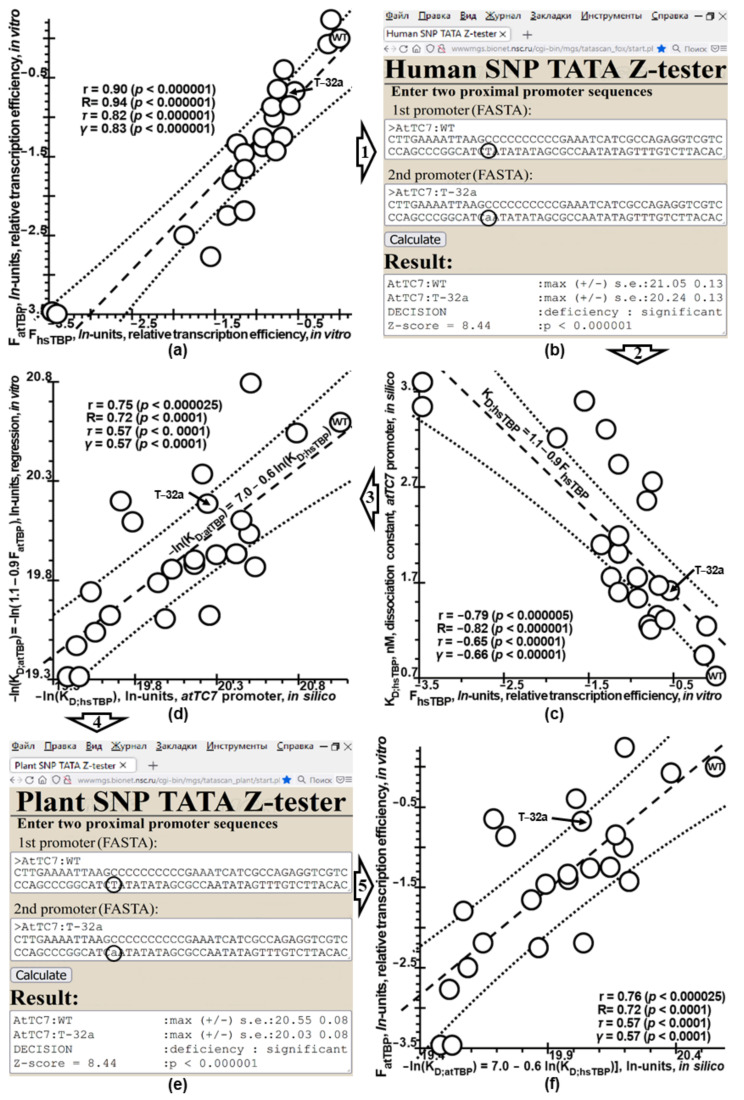
A stepwise flowchart of an empirical solution (found in this work) to the ill-posed inverse problem of how to uniformly estimate the effects of mutations in plant proximal promoters on gene expression. *Legend*: see the legend of Figure 1. (**a**) The interchangeability of hsTBP (*x*-axis) and atTBP (*y*-axis) for transcription in vitro [45]. (**b**) Using Human_SNP_TATA_Z-tester [28] (see in-depth description in the Supplementary Materials [18,28,29,30,31,32,33,35,36,37,38,39,41,42,62,63,64,65,66,67]), we estimated the −ln(K_D; hsTBP_) values of hsTBP affinity for the 90 bp DNA sequence of either the wildtype or the mutant variant of the *Agrobacterium tumefaciens* T-DNA TC7 promoter (*AtTC7*) [54] from [45], as shown by arrow 1. (**c**) Statistically significant linear regression of the predicted K_D;hsTBP_ values in relation to the F_hsTBP_ values measured using hsTBP [45], as expressed by Equation (1) written above the dashed line. (**d**) Statistically significant linear regression of ln(K_D;atTBP_) values of atTBP affinity for the promoters in question, which are the F_atTBP_ values of the reporter gene expression measured in vitro using atTBP [45] and, here, rescaled using Equation (1) via the (calculated above) −ln(K_D;hsTBP_) values of hsTBP affinity for the same promoters, as a solution to the ill-posed inverse problem under study, as expressed by Equation (2) written above the dashed line. (**e**) The Plant_SNP_TATA_Z-tester Web service created in this work implements the abovementioned solution to the ill-posed inverse problem being analyzed. (**f**) Statistically significant correlations between the −ln(K_D;atTBP_) values calculated by Plant_SNP_TATA_Z-tester and measured effects of the mutations in the plant proximal promoter in question on gene expression, using atTBP in vitro [45]. Within Tikhonov’s regularization [61], these correlations characterize the match between the approximate solution found in this work to the ill-posed problem under study and its unknown true solution.

That is why, not being able to find the exact solution to the ill-posed inverse problem in plants, we constructed an approximate solution using Tikhonov’s regularization [61]. Figure 2a shows the statistically significant correlations between datasets 1 and 2 (Table 1) corresponding to in vitro gene expression driven by thale cress TBP (atTBP) and human TBP (hsTBP) binding to the eukaryotic TC7 promoter from the T-DNA region of the Ti plasmid of oncogenic *Agrobacterium tumefaciens* strains [45]. These strains are capable of infecting both plants [54] and humans [55,56]. 

Within Tikhonov’s regularization [61], this correlation (Figure 2a) characterizes a similarity between the ill-posed inverse problem of evaluating the transcriptional outcome of mutations in plant proximal promoters (Figure 1) and the analogous well-posed problem for humans, which has already been solved using our public Web service Human_SNP_TATA_Z-tester [28] (see in-depth description in the Supplementary Materials [18,28,29,30,31,32,33,35,36,37,38,39,41,42,62,63,64,65,66,67]). With this in mind, Figure 2 depicts how we adapted it step-by-step for comparing between wildtype and mutant variants of the plant promoter DNA sequences under study in their effects on gene expression, i.e., our new Web service Plant_SNP_TATA_Z-tester created in this work. 

At the first step (Figure 2: arrow 1), we analyzed each of the 24 variants of the T-DNA TC7 promoter [45] (Table 1: datasets 1 and 2) using our Web service Human_SNP_TATA_Z-tester (Figure 2b) to obtain −ln(K_D;hsTBP_), i.e., the dissociation constant for hsTBP expressed in the natural logarithm units (ln units). 

At the second step (Figure 2: arrow 2), we rescaled these values from ln units into nanomoles per liter (nM), which strongly and statistically significantly correlated with the relative transcription efficiency rates, which were experimentally determined for hsTBP (F_hsTBP_) [45] (Figure 2c). The corresponding linear regression is given by

K_D;hsTBP_ = 1.1 − 0.9F_hsTBP_. (1)

At the third step (Figure 2: arrow 3), because hsTBP and atTBP are interchangeable with each other under the in vitro experimental conditions [45], we substituted the relative transcription efficiency rates experimentally determined for atTBP (F_atTBP_) into Equation (1) instead of F_hsTBP_ to estimate the affinity of recombinant atTBP for the same variants of the TC7 promoter (i.e., K_D;atTBP_ values) and then rescaled them to ln units, as shown on the *y*-axis in Figure 2d. Correlating −ln(K_D,atTBP_) with previously calculated −ln(K_D,hsTBP_) yielded a statistically significant linear regression, i.e.,

−ln(K_D;atTBP_) = 7.0 − 0.6ln(K_D;hsTBP_). (2)

Thus, Equation (2) represents the target model, which made it possible to create the public Web service Plant_SNP_TATA_Z-tester (Figure 2e); the latter integrates the model underlying our Web service Human_SNP_TATA_Z-tester [28] with Equation (2) at the fourth step (Figure 2: arrow 4).

At the final step (Figure 2: arrow 5), we compared the output of Plant_SNP_TATA_Z-test with the experimental data about TC7-driven transcription initiated by recombinant atTBP [45], as depicted in Figure 1f. This step uncovered a statistically significant correlation between them, which, within Tikhonov regularization [61], characterizes how much the approximate solution of the ill-posed problem designed in this work fits an unknown true solution of this problem. 

### 2.3. Determining Application Limits of Plant_SNP_TATA_Z-Tester by Means of Experimental Data on Tobacco Development in the Dark or under Light, Indicating That the Inverse Problem under Study Is Ill-Posed

**Figure 3:**
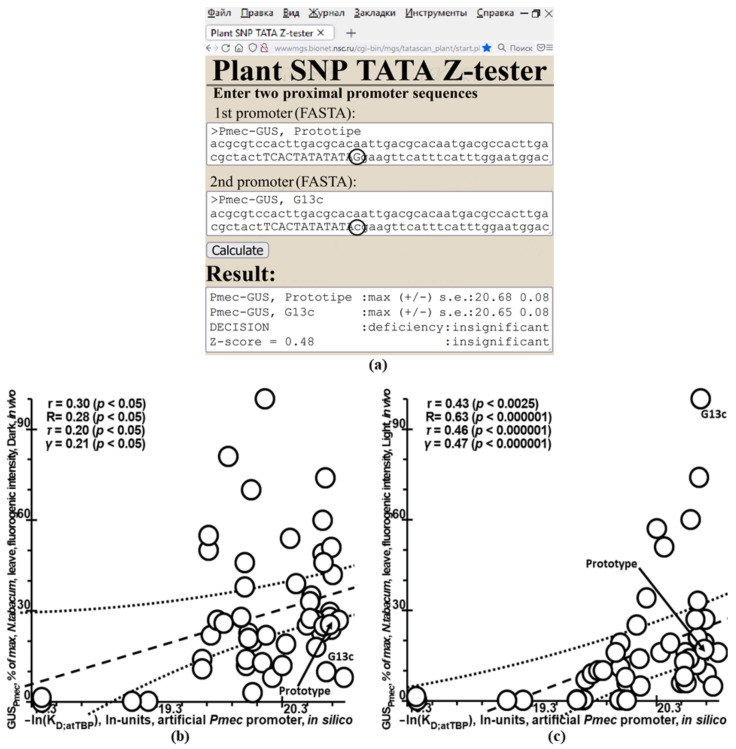
Determining the application limits of Plant_SNP_TATA_Z-tester on experimental data about tobacco development in the dark or under light [53], which indicated that the inverse problem under study is ill-posed. *Legend*: see the legend of Figure 1. (**a**) The result of our Web service Plant_SNP_TATA_Z-tester created in this work in the case of the comparison between the prototype *Pmec* of the artificial promoter for plant genes (the textbox “1st promoter”) and its mutant variant “G13c” (the textbox “2nd promoter”). (**b**,**c**) Statistically significant correlations between the in silico predicted −ln(K_D_) values of TBP–promoter affinity expressed in ln units, which characterize the complexes formed by tobacco TBP binding to various artificial promoters based on the *Pmec* prototype [59] (*x*-axis) and the in vivo efficiency magnitudes of the reporter gene *gusA* expression (*y*-axis) on tobacco development in the dark or under light, respectively.

First of all, we determined the application limits of Plant_SNP_TATA_Z-tester using experimental data on tobacco development in the dark or under light (Table 1: datasets 11 and 12) [53], which determined the ill-posed nature of the inverse problem (Figure 1). To this end, we applied Plant_SNP_TATA_Z-tester to compare the prototype *Pmec* (the textbox “1st promoter” in Figure 3a) with each mutant variant (the textbox “2nd promoter” in this figure) pairwise, as exemplified by variant “G13c”. As a result, we obtained the in silico predicted −ln(K_D_) values of the TBP–promoter affinity expressed in ln units, depending on a *Pmec* variant, as plotted along the *x*-axis in Figure 3b,c. 

Next, we correlated these values with in vivo transcription efficiencies of the *gusA* reporter gene. Remarkably, this analysis resulted in statistically significant correlations between the in silico predicted and the in vivo measured effects of mutations on the reporter gene expression for both dark- and light-grown plants (Figure 3b,c, accordingly). 

These correlations reflect the conventional viewpoint that TBP-dependent formation of the transcription preinitiation complex in place of the transcriptionally inactive core-promoter nucleosome is the obligatory step within the multistep eukaryotic gene expression machinery [68]. Thus, mutations altering the TBP-binding sites within plant promoters can autonomously modulate gene expression regardless of binding sites for other regulatory proteins unless these mutations also change them, as proven experimentally in *Saccharomyces cerevisiae* [15]. 

At the same time, TBP–DNA affinity by itself (i.e., the predicted dissociation constant) could explain only ~10% of gene expression variation observed in tobacco plants under different experimental conditions (development in the dark or under light). This finding is indicative of a significant contribution of other transcriptional regulators (e.g., transcription factors) to in vivo gene expression alteration driven by SNPs near the TBP-binding sites within the proximal promoters in plants. 

This line of reasoning determines the application limitations of Plant_SNP_TATA_Z-tester created here.

### 2.4. Verification of Plant_SNP_TATA_Z-Tester Using Independent Experimental Data on Mutations within Natural Proximal Promoters of Plant Genes

Next, we evaluated Plant_SNP_TATA_Z-tester using independent experimental data on the mutations within natural proximal promoters of plant genes (Table 1: datasets ## 3–8). 

**Figure 4:**
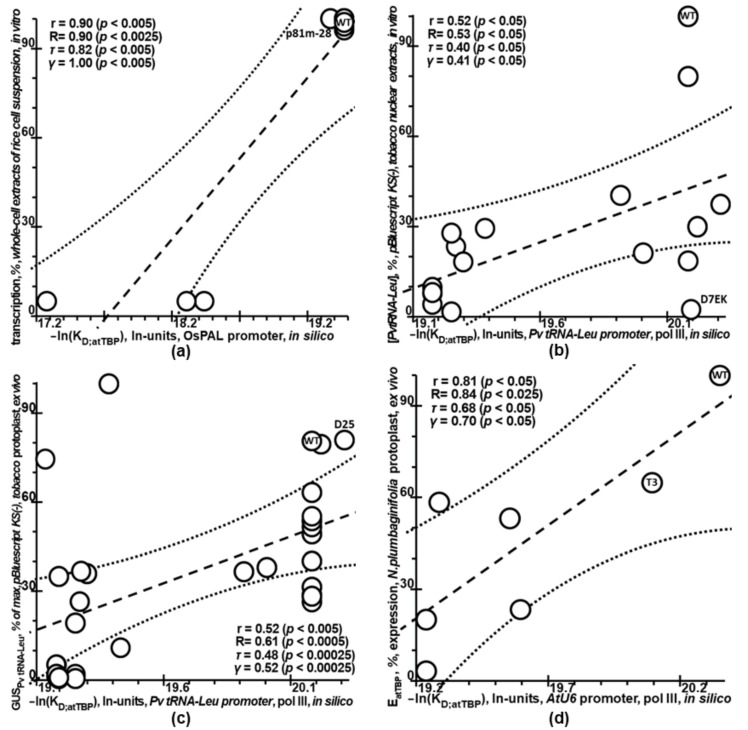

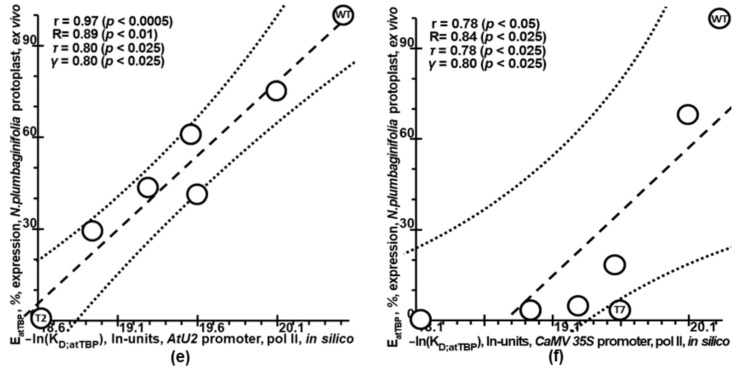
Verification of Plant_SNP_TATA_Z-tester using experimental data on the natural plant promoters (datasets 3–8). (**a**) The rice gene *PAL* promoter (phenylalanine ammonia-lyase) and Pol II, in whole-cell extracts of rice cell suspension cultures in vitro [46,47]. (**b**,**c**) The bean *tRNA-Leu* gene prompter, Pol III, in vitro [48] and ex vivo [49], respectively. (**d**) The thale cress U6-26 snRNA gene promoter (U6 small nuclear RNA; TAIR ID AT3G13855 [57]) and Pol III [50]. (**e**) The thale cress U2.2 snRNA gene promoter (U2 small nuclear RNA; TAIR ID AT3G57645 [57]). (**f**) The cauliflower mosaic virus (CaMV) promoter for the viral 35S transcript (GenBank AC MT611510 [58]), (**e**,**f**) RNA polymerase II. (**d**–**f**) Tobacco protoplasts ex vivo [50]. The natural (WT) and one of the mutant variants of the promoter under study are indicated.

In Figure 4, readers can see statistically significant correlations between the experimentally measured effects of mutations in plant promoters on gene expression and those predicted by Plant_SNP_TATA_Z-tester. These correlations are resistant to variation of the correlation criteria tested, of the plant natural promoters subjected to mutagenesis, and of experimental conditions (in vitro and ex vivo). Thus, although Plant_SNP_TATA_Z-tester is an approximate solution to the ill-posed inverse problem of estimating the effects of mutations in the T-DNA TC7 promoter on gene expression in vitro [45], it provides reliable estimates for a wider range of experimental systems.

### 2.5. Validation of Plant_SNP_TATA_Z-Tester by Means of Experimental Data on Mutations in the Synthetic Proximal Promoters Designed on the Basis of Natural Ones

**Figure 5:**
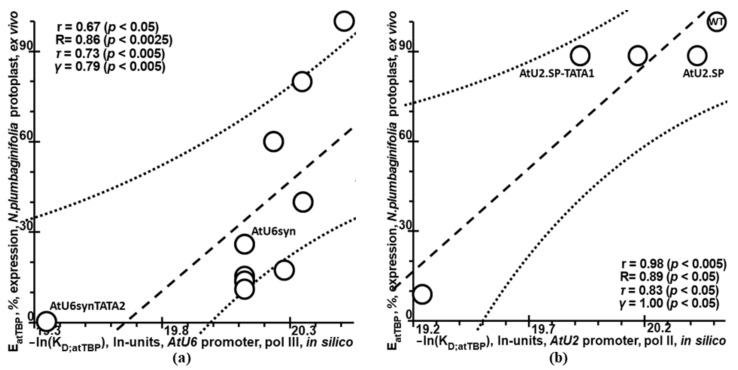
Verification of Plant_SNP_TATA_Z-tester using experimental data on the plant gene synthetic artificial proximal promoters designed on the basis of natural ones (datasets 9 and 10). (**a**) The synthetic artificial promoter designed on the basis of the thale cress U6-26 snRNA gene promoter, Pol III, and tobacco protoplasts ex vivo [51]. (**b**) The synthetic artificial promoter designed on the basis of the thale cress U2.2 snRNA gene promoter, Pol II, and tobacco protoplasts ex vivo [52].

Additionally, we assessed Plant_SNP_TATA_Z-tester using independent experimental data on mutations in synthetic artificial proximal promoters designed on the basis of natural ones (Table 1: datasets 9 and 10; Figure 5). A comparison of Figure 4 and Figure 5 indicates the uniformity of the results of our Web service Plant_SNP_TATA_Z-tester when mutations were evaluated both in natural promoters of plant genes and in synthetic artificial promoters designed by analogy with natural ones, respectively. 

## 3. Discussion

**Figure 6:**
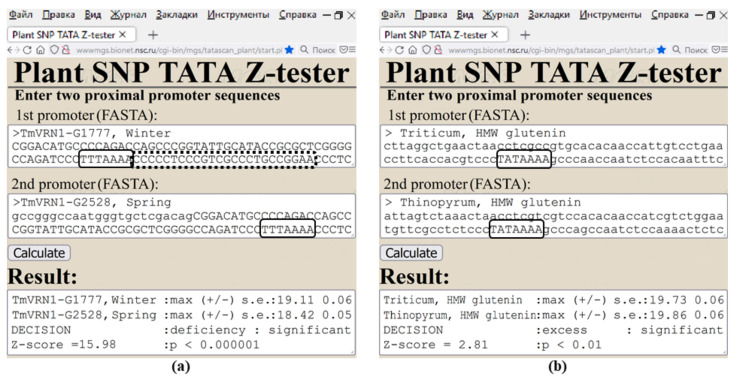
Examples of the output of Web service Plant_SNP_TATA_Z-tester regarding assessment of agriculturally valuable mutations in plant proximal promoters for gene expression responsive to environmental factors during wheat development. (**a**) Deletions of the spacer (dotted box) between a TBP-binding site (solid box) and a TSS of wheat gene *VRN1* can downregulate vernalization protein 1 encoded by this gene, which is the widely accepted genome-wide molecular marker of spring wheats in contrast to winter wheats [69]. (**b**) Statistically significant upregulation of the glutenin high-molecular-weight (HMW) subunit, which determines the gluten level in the grain, in wheatgrass (*Thinopyrum*) as compared to wheat (*Triticum*) [70]. This result explains how, in the harsh Siberian climate, wheat–wheatgrass hybrids increase grain baking quality without yield losses in comparison with the mother wheat variety [71].

As a discussion of the results of our freely available Web service Plant_SNP_TATA_Z-tester, Figure 6 presents how it actually assesses agriculturally valuable mutations in plant promoters [69,70,71]. First of all, deletions of the spacer between a TBP-binding site and TSS of the wheat gene *VRN1* can downregulate vernalization protein 1 encoded by this gene, representing the conventional genome-wide molecular marker of spring wheats in contrast to winter wheats [69]. 

Thus, at the molecular level, some SNPs near TBP-binding sites of promoters of the most crucial plant genes can denote agriculturally valuable strains, whereas, on the whole-genome scale, the contribution of the gene expression alterations (responsive to environmental factors during plant development) to intraspecific diversity can exceed such a total contribution of all SNPs in the plant gene promoters (Figure 3b,c).

At last, with respect to wheat (*Triticum*), wheatgrass (*Thinopyrum*) as the most tenacious malicious hard-to-eradicate weed in Siberia can statistically significantly overexpress the glutenin high-molecular-weight subunit determining the gluten level in the grain [70]. This may explain how wheat–wheatgrass hybrids increase grain baking quality without yield losses in the harsh Siberian climate in comparison with the mother wheat variety [71]. Thus, our public Web service Plant_SNP_TATA_Z-tester created in this work is suitable for designing targeted hybridization of cultivated plants with their wild congeners [6] as the oldest approach in mainstream postgenomic agriculture [5], along with synthetic pinpoint nature-like optimization of promoters for plant genes [3] and both QTL- and SNP marker-assisted breeding [7].

## 4. Materials and Methods 

### 4.1. Experimental Data under Study

In this work, we analyzed all the publicly available independent experimental data—on the effects of mutations in plant proximal promoters on gene expression—that we could find within the PubMed database [44], as listed in Table 1. A total of 242 wildtype or mutant variants of plant promoters are presented using 90 bp DNA sequences upstream of TSSes of the reporter genes along with quantitative magnitudes of expression of these genes under the experimental conditions cited in the rightmost column of this table.

### 4.2. In Silico Analysis of DNA Sequences

We processed DNA sequences by means of our public Web service Plant_SNP_TATA_Z-tester (e.g., Figure 3a and Figure 6) created in this work, as depicted in Figure 2. To this end, as its prototype, we used our previously developed Web service Human_SNP_TATA_Z-tester [28] (see description [28,29,30,31,32,33,35,36,37,38,39,41,42,62,63,64,65,66,67] in the Supplementary Materials), and we expanded it only with Equation (2) in line with Tikhonov’s regularization [61].

### 4.3. The Knowledge Base (on Effects of Mutations in Plant Promoters on Gene Expression) Created in this Work

For each dataset listed in Table 1, by means of the 90 bp DNA sequences of the mutant versus wildtype plant promoters, we predicted the effects of mutations on the reporter gene expression using Plant_SNP_TATA_Z-tester, as exemplified in Figure 2, Figure 3, Figure 4, Figure 5 and Figure 6. Next, we documented these predictions together with the corresponding experimental measurements as a textual flat file in an Excel-compatible format. Lastly, in the MariaDB 10.2.12 Web environment (MariaDB Corp AB, Espoo, Finland), we added this document to our knowledge base Plant_SNP_TATAdb (created in this work), whose pilot version is freely available at https://www.sysbio.ru/Plant_SNP_TATAdb/, accessed on 10 June 2022.

### 4.4. Statistical Analysis

For each dataset listed in Table 1, using the Statistica software (Statsoft^TM^, Tulsa, OK, USA), we conducted analyses of Pearson’s linear correlation, Spearman’s rank correlation, Kendall’s rank correlation, and Goodman–Kruskal generalized correlation between the experimentally measured effects of mutations in plant proximal promoters on gene expression and those predicted by our Web service Plant_SNP_TATA_Z-tester created in this work, as shown in Figure 1, Figure 2, Figure 3, Figure 4 and Figure 5.

## 5. Conclusions

In this work, for the first time, we compiled all the independent experimental data (Table 1)—applicable to the investigation into how mutations in plant proximal promoters can affect gene expression—that we could find in the PubMed database [44]. Although these data cast doubt on the very possibility of unequivocally estimating the impact of proximal promoter mutations on plant gene expression (Figure 1), due to the use of Tikhonov’s regularization for ill-posed problems (Figure 2) [61], we managed to create our public Web service Plant_SNP_TATA_Z-tester, whose predictions correlated statistically significantly and robustly with all experimentally measured effects of mutations in plant proximal promoters on gene expression (Figure 3, Figure 4 and Figure 5). Accordingly, we exemplified how it can actually rate agriculturally valuable mutations in plant proximal promoters (Figure 6). For this reason, we can conclude that there is some hope that practical use of this tool may reduce the labor, cost, and time required for the progress of mainstream postgenomic agriculture [5], including synthetic pinpoint nature-like optimization of plant gene promoters [3], targeted design of hybrids of cultivated plants with their wild congeners [6], and both QTL- and SNP marker-assisted breeding [7].

## Figures and Tables

**Table 1:** The experimental data—on the effects of mutations in the plant promoters on gene expression—that we could find in the PubMed database [44].

Dataset #	Promoter	Transcribed Gene	Conditions	TBP	Pol	*N*	Ref.
1	TC7	RNA transcript template of G-free sequence	In vitro: standard transcription reaction	Human	II	24	[45]
2			A chimeric in vitro system in which human TATA-binding protein (hsTBP) was replaced by purified TBP-1 of thale cress (atTBP)	Thale cress (*Arabidopsis thaliana*)		24	
3	*OsPAL*	*OsPAL*	In vitro: whole-cell extracts of rice cell suspension cultures	Rice (*Oryza sativa)*	II	8	[46,47]
4	*Pv tRNA-Leu*	*Pv tRNA-Leu*	In vitro: tobacco cell nuclear extract	Tobacco (*Nicotiana plumbaginifolia*)	III	16	[48]
5	*Pv tRNA-Leu*	*gusA*	Ex vivo: transient expression in tobacco protoplasts		III	30	[49]
6	*AtU6-26 snRNA*	*AtU6-26 snRNA*			III	7	[50]
7	*AtU2.2 snRNA*	*AtU2.2 snRNA*			II	7	
8	*CaMV 35S*	*CaMV 35S*			II	7	
9	synthetic promoters based on *AtU6snRNA*	*At U6-26 snRNA*			III	10	[51]
10	synthetic promoters based on *AtU2snRNA*	*At U2.2 snRNA*			II	5	[52]
11	*Pmec*	*gusA*	In vivo: dark-grown tobacco leaves		II	52	[53]
12			In vivo: light-grown tobacco leaves		II	52	
TOTAL	10 promoters	7 reporter genes	6 experimental systems	4 TBPs	2 Pols	242	9 Refs

Note: Pol II and III: RNA polymerases II and III, respectively. *N*: the number of variants of a promoter DNA sequence, each of which were quantitatively characterized in terms of their effects on gene expression. TC7: a eukaryotic promoter within the T-DNA region of the Ti plasmid of oncogenic *Agrobacterium tumefaciens* strains, which infect plants [54] and humans [55,56]. *gusA*: the gene encoding β-glucuronidase; *OsPAL*: the rice (*Oryza sativa*) gene encoding phenylalanine ammonia-lyase. *Pv tRAN-Leu:* the bean (*Phaseolus vulgaris*) tRNA-Leu gene promoter. *At U2.2 snRNA* and *At U6-26 snRNA*: the thale cress (*Arabidopsis thaliana*) genes encoding U2 and U6 small nuclear RNAs [57], respectively. *CaMV 35S*: cauliflower mosaic virus promoter for the 35S viral transcript [58]; *Pmec*: the artificial plant-addressed promoter [59].

## Supplementary Materials

The supporting information can be downloaded at www.mdpi.com/article/10.3390/ijms23158684/s1. References [18,28,29,30,31,32,33,35,36,37,38,39,41,42,62,63,64,65,66,67] are cited in the Supplementary Materials.

## Abbreviations

CaMV	Cauliflower mosaic virus
HMW	High-molecular-weight
ln units	Natural logarithm units
QTL	Quantitative trait loci
PAM	Protospacer-adjacent motif
SNP	Single-nucleotide polymorphism
TAIR	The *Arabidopsis* Information Resource
TBP	TATA-binding protein
TSS	Transcription start site
