# Supplementary material for: Plant_SNP_TATA_Z-Tester: A Web Service That Unequivocally Estimates the Impact of Proximal Promoter Mutations on Plant Gene Expression
Source: Int J Mol Sci. 2022 Aug 4;23(15):8684. doi: 10.3390/ijms23158684 (PMC9369029; doi:10.3390/ijms23158684)
Supplement: Supplementary file 1 [file ijms-23-08684-s001.zip › ijms-1818824-supplementary.pdf]

International Journal of *Molecular Sciences*

MDPI

Supplementary Materials

Plant_SNP_TATA_Z-tester: a Web Service that Unequivocally Estimates the Impact of Proximal Promoter Mutations on Plant Gene Expression

Dmitry Rasskazov[1], Irina Chadaeva[1], Ekaterina Sharypova[1], Karina Zolotareva[1], Bato Khandaev[1], Petr Ponomarenko[1], Nikolay Podkolodnyy[1,2], Natalya Tverdokhleb[1], Oleg Vishnevsky[1], Anton Bogomolov[1], Olga Podkolodnaya[1], Ludmila Savinkova[1], Elena Zemlyanskaya[1], Vadimir Golubyatnikov[3], Nikolay Kolchanov[1], Mikhail Ponomarenko[1,*]

1 Institute of Cytology and Genetics, Novosibirsk 630090, Russia

2 Institute of Computational Mathematics and Mathematical Geophysics, Novosibirsk 630090, Russia

3 Sobolev Institute of Mathematics, Novosibirsk 630090, Russia

* Correspondence: pon@bionet.nsc.ru. Tel.: +7 (383) 363-4963 ext. 1311 (M.P.)

Human_SNP_TATA_Z-tester as the prototype used in this work to empirically solve an ill-posed inverse problem about how to uniformly estimate effects of mutations in proximal promoters on gene expression in plant grown under various environmental conditions during development (Figure 2b)

For our previously developed Web-service Human_SNP_TATA_Z-tester [28] shown in Figure 2b (hereinafter, see the main text), the input is two 90 bp DNA sequences, which are $S_{wt} = \{s^{wt}_{-90} \ldots s^{wt}_{i} \ldots s^{wt}_{-1}\}$ and $S_m = \{s^{min}_{-90} \ldots s^{min}_{i} \ldots s^{min}_{-1}\}$ immediately before the transcription start site (TSS, $s^{wt}_0 = s^{min}_0$, where: $s^{\bullet}_i \in \{a, c, g, t\}$) of the human gene proximal promoter carrying either ancestral (wt) or minor (min) allele, respectively, of an arbitrary SNP under study. In this figure, readers can see them in two textboxes "1st promoter" and "2nd promoter", respectively.

After clicking the "Calculate" button (Figure 2b), using our three-step model [29] of the TBP-promoter binding (i.e., TBP slides along DNA [18] ↔ molecular co-recognition between TBP and TBP-site met [30] ↔ DNA-bend stabilizes TBP-promoter complex [32]) as proven experimentally [33], our Web-service Human_SNP_TATA_Z-tester [28] estimated two "$-\ln[K_D(S_\bullet)]$"-values expressed on the natural-logarithm scale (ln-units), which evaluate the TBP-promoter affinity upon each DNA sequences ($S_\bullet$) independently from one another, such as:

$$-\ln[K_D(S_\bullet)] = 10.9 - 0.2\,\{\ln[K_{SLIDE}(S_\bullet)K_{STOP}(S_\bullet)K_{BEND}(S_\bullet)]\}, \qquad \textbf{(S1)}$$

where K_D is the equilibrium dissociation constant estimation (in moles per liter, M); 10.9 (ln-units) seems to numerically match nonspecific TBP–DNA affinity (10 μM) as measured independently [62]; 0.2 is a stoichiometric coefficient of the three-step TBP–promoter binding, as determined elsewhere by means of the difference in the length of the TBP consensus site and the region of TBP sliding along DNA [29].

First of all, within Eq. S1, $-\ln[K_{STOP}(S_\bullet)]$ is an estimation of the equilibrium dissociation constant of the mutual recognition between TBP and the most probable TBP-site encountered at the second step among the three steps in question:

$$-\ln[K_{STOP}(S_\bullet)] = MAX_{-90 \le i \le -20;\ k \in \{-1; +1\}}\{\Sigma_{i-1 \le j \le i+13}\ w\{i, s^{\bullet}_{j;k}\}, \qquad \textbf{(S2)}$$

where $w\{i, s^{\bullet}_j\}$ is Bucher's weight of nucleotide $s^{\bullet}_j$ at the *j*th position of the TBP-site [31]; k is an indicator of either a direct (+1) or complementary (−1) strand of the double-stranded B-helical DNA of the promoter under study.; MAX(ζ) is the highest ζ-value observed.

Besides, in Eq. S1, $-\ln[K_{SLIDE}(S_\bullet)]$ is an estimate of the equilibrium dissociation constant of an interaction between TBP and the promoter DNA during their sliding one over the other at the first step among the three within this bioinformatics model, as:

$$-\ln[K_{SLIDE}(S_\bullet)] = MEAN_{[\,\xi-7;\,\xi+19];\ k\in\{-1;+1\}}\,(35.1\mu + 0.8[TA]), \quad \textbf{(S3)}$$

where ξ is the position of the most probable TBP-site according to Bucher's criterion [31] (i.e., Eq. S2); the μ value of the minor-groove width of the B-helical DNA at this site's center was determined elsewhere [63]; [TA] is the concentration of dinucleotide TA; 0.8 and 35.1 are linear regression coefficients [64].

Finally, in Eq. S1, $-\ln[K_{BEND}(S_\bullet)]$ is an estimation of the equilibrium dissociation constant of intermediate short-lived complexes between TBP and each of two DNA strands of the TBP-site separately from one another during DNA melting leading to the bend that fixes the TBP–promoter complex [32] at the last step of their binding, as follows:

$$-\ln[K_{BEND}(S_\bullet)] = MEAN_{[\,\xi-7;\xi+19];\ k\in\{-1;+1\}}\,(0.9[TA, AA, TG, AG] + 2.5[TA, TC, TG] + 14.4), \quad \textbf{(S4)}$$

where 0.9, 2.5, and 14.4 are linear regression coefficients [64].

After that, examining all the possible mutations, $s^\bullet_j \rightarrow \varphi$, at each *j*th position among 26 positions of the most probable TBP-site according to Eq. S2, our Web-service Human_SNP_TATA_Z-tester [28] estimated standard error of the mean SEM● of the $-\ln[K_D(S_\bullet)]$ values calculated using Eq. S1, as:

$$SEM_\bullet = \{(\Sigma_{\xi-7\le j\le \xi+19}\Sigma_{\varphi\in\{a,c,g,t\}}\ln[K_D(s^\bullet_{\xi-7}\ldots s_{j-1}\varphi s_{j+1}\ldots s^\bullet_{\xi+19}) / K_D(s^\bullet_{\xi-7}\ldots s_{j-1}s_j\, s_{j+1}\ldots s^\bullet_{\xi+19})]^2\} / ((3{*}26)(3{*}26-1))]^{1/2}. \quad \textbf{(S5)}$$

Using both sequences S_{wt} and S_{min} and Eqs. S1 – S5, this toolbox found two paired value sets $\{-\ln[K_D(S_{wt})] \pm SEM_{wt}\}$ and $\{-\ln(K_D(S_{min})) \pm SEM_{min}\}$, respectively, which are necessary for Fisher's Z-score [65], for instance:

$$Z = abs\{\ln[K_D(S_{wt}) / K_D(S_m))] / [SEM_{wt}^2 + SEM_m^2\}^{1/2}.. \quad \textbf{(S6)}$$

Eventually, with the help of the R software [65], using this Z-value, our Web-service Human_SNP_TATA_Z-tester [28] found a *p* value of the probability of the tested hypothesis "H_0: $K_D(S_{wt}) \neq K_D(S_{min})$" so that if it is statistical significant ($p > 0.95$), it made the decision:

IF {**INEQUALITY** "$K_D(S_{min}) < K_D(S_{wt})$" is statistically significant },
THEN {**PREDICTION** is "the minor allele of the gene considered is overexpressed relative to the ancestral one"};
ELSE [**IF** {**INEQUALITY** "$K_D(S_m) > K_D(S_{wt})$" is statistically significant}, **(S7)**
THEN {**DECISION** is "the minor allele of this gene is underexpressed relative to the ancestral one"},]
OTHERWISE {**DECISION** is "the expression change of this gene is insignificant"}.

Our Web service Human_SNP_TATA_Z-tester [28] presents this decision (Eq. S7) in the "Decision" line of the "Result" textbox, while all the intermediate results are in the other lines of this textbox, as readers can see in Figure 2b. Table S1 presents how we experimentally selectively confirmed this from article to article [28, 35-39, 41, 42, 66, 67]. Figure S1 does this in graphical form.

Table S1. An electrophoretic mobility shift assay (EMSA)-based in vitro verification of the complex of TBP with synthetic 26 bp oligodeoxyribonucleotides (ODNs) identical to natural human gene promoters near each SNP tested

#	*Human gene*, **dbSNP ID [66]**	**WT min**	**26 bp oligodeoxyribonucleotides, direct strand 5′-ODN-3′**	Prediction, ln units		Experiment, *in vitro*, ln units		
				$-\ln(K_D)$	$\Delta\ln(K_D)$	$-\ln(K_D)$	$\Delta\ln(K_D)$	**Reference**
1	*LEP*	WT	gatcgggcc**G**CT**A**TAAG**A**ggggcggg	19.43		16.37		[35]
2	rs34104384	A−30T	gatcgggccGCTATAAG**T**ggggcggg	19.70	0.27	16.43	0.06	
3	rs201381696	A−35G	gatcgggccGCT**G**TAAGAggggcggg	18.22	−1.21	15.28	−1.09	
4	rs200487063	G−38A	gatcgggcc**A**CTATAAGAggggcggg	19.85	0.42	16.96	0.59	
5	*ABCA9*	WT	aattatttg**T**ATATTTctgagcatac	19.66	−0.88	16.47	−0.88	[39]
6	rs367781716	T−37C	aattatttg**C**ATATTTctgagcatac	18.78		15.59		
7	F9	WT	tttggTACAA**C**TAATcgaccttacca	18.86	0.46	15.05	1.17	[41]
8	rs750827465	C-34A	tttggTACAA**A**TAATcgaccttacca	19.32		16.22		

Table S1. *Cont.*

#	*Human gene,* dbSNP ID [66]	WT min	26 bp oligodeoxyribonucleotides, direct strand 5′-ODN-3′	Prediction, ln units		Experiment, *in vitro*, ln units		
				-ln(K_D)	Δln(K_D)	-ln(K_D)	Δln(K_D)	Reference
9	*HBD*	WT	acaggaccagC**AT**AAAAggcagggca	19.29		17.14		[36]
10	rs34166473	T−30C	acaggaccagCA**T**AAAAggcagggca	18.27	−1.02	15.02	−2.12	
11	rs35518301	A−31G	acaggaccagC**G**TAAAAggcagggca	18.65	−0.64	16.12	−1.02	[42]
12	*MBL2*	WT	catctatttcTA**T**ATAGcctgcaccc	20.17	−0.87	17.20	-0.47	
13	rs72661131	T−39C	catctatttcTA**C**ATAGcctgcaccc	19.30		16.73		
14	*IL1B*	WT	ttttgaaagc**C**ATAAAAacagcgagg	19.22	0.94	17.73	1.42	
15	rs1143627	C−31T	ttttgaaagc**T**ATAAAAacagcgagg	20.16		19.15		
16	*TPI1*	WT	cgcggcgctcTATA**T**AAgtgggcagt	20.70	−1.41	19.31	−3.13	
17	rs1800202	T−26G	cgcggcgctcTATA**G**AAgtgggcagt	19.29		16.12		
18	*F3*	WT	gccggcccTTTATAg**c**gcgcggggca	19.60	0.42	16.45	1.02	
19	rs563763767	c−21T	gccggcccTTTATAg**T**gcgcggggca	20.02		17.47		
20	*HBB*	WT	cagggctggg**C**A**TAAAA**gtcagggca	19.20		16.81		
21	rs34598529	A-28G	cagggctgggCATA**G**AAgtcagggca	18.34	-0.86	14,40	-2.41	
22	rs34598529	A-28C	cagggctgggCATA**C**AAgtcagggca	18.63	-0.57	14.51	-2.30	
23	rs281864525	A-25C	cagggctgggCATAAA**C**gtcagggca	18.73	-0.47	15.71	-1.10	
24	rs63750953	Δ-25AA	ccagggctgggCATA***Ag***tcagggcag	18.61	-0.59	15.71	-1.10	
25	rs33980857	T-29A	cagggctgggCA**A**AAAAgtcagggca	17.70	-1.50	16.02	-0.79	
26	rs33980857	T-29C	cagggctgggCA**C**AAAAgtcagggca	18.17	-1.03	15.78	-1.03	
27	rs33980857	T-29G	cagggctgggCA**G**AAAAgtcagggca	17.67	-1.53	16.02	-0.79	
28	rs33931746	A-27T	cagggctgggCATA**T**AAgtcagggca	19.75	0.55	16.63	-0.18	
29	rs34598529	A-28G	cagggctgggCAT**G**AAAgtcagggca	17.85	-1.35	14.51	-2.30	
30	rs34500389	C-32T	cagggctggg**T**ATAAAAgtcagggca	20.18	0.98	17.26	0.45	[37]
31	*HBZ*	WT	agctccctgTA**T**ATAAggggaccctg	20.76	−1.48	18.93	−1.90	
32	rs11318094	T-29A	agctccctgTA**A**ATAAggggaccctg	19.28		17.03		
33	*EPOR*	WT	cacgtcat**c**T**A**T**T**TTTGTctgctacg	18.24		15.05		
34	rs1006576690	T-27A	cacgtcatcTAT**A**TTTGTctgctacg	19.39	1.15	18.33	2.74	
35	rs971717705	A-29G	cacgtcatcT**G**TTTTTGTctgctacg	18.11	-0.13	15.71	0.12	
36	rs567946217	c-31A	cacgtcat**A**TATTTTTGTctgctacg	19.33	1.09	17.68	2.09	
37	rs567946217	c-31G	cacgtcat**G**TATTTTTGTctgctacg	18.51	0.27	16.34	0.75	
38	*GCG*	WT	gctggagagT**A**TATAAAAgcagtgcg	20.89	−0.61	18.64	−0.81	[38]
39	rs183433761	A−41G	gctggagagT**G**TATAAAAgcagtgcg	20.28		17.83		

Table S1. *Cont.*

#	*Human gene,* dbSNP ID [66]	WT min	26 bp oligodeoxyribonucleotides, direct strand 5′-ODN-3′	Prediction, ln units		Experiment, *in vitro*, ln units		
				-ln(KD)	Δln(KD)	-ln(KD)	Δln(KD)	Reference
40	*ASMT*	WT	ggtgaccttttgt**G**cccagaataggt	18.18	0.75	14.33	−0.51	[28]
41	rs1402972626	G−30A	ggtgaccttttgt**A**cccagaataggt	18.93		13.82		
42	*CDY2A*	WT	agaatgttccata**T**aatcgtcatagc	19.27	−0.51	15.65	−1.14	
43	rs20067072	T−24C	agaatgttccata**C**aatcgtcatagc	18.76		14.51		
44	*GTPBP6*	WT	atcacgagcacgt**G**atgaggagcggc	17.30	1.38	13.41	0.07	
45	rs1393008234	G−24T	atcacgagcacgt**T**atgaggagcggc	18.68		13.48		
46	*SHOX*	WT	gaggtcgccgcgt**A**taaatagtgaga	20.31	−1.10	17.06	−1.90	
47	rs1452787381	A-45G	gaggtcgccgcgt**G**taaatagtgaga	19.21		15.16		
48	*ZFY*	WT	ggcggaggggggcc**C**aactaccatccc	17.67	0.51	13.82	−0.70	
49	rs1452787381	C−56T	ggcggaggggggcc**T**aactaccatccc	18.18		13.12		
50	*GRIN1*	WT	tggagg**g**ggACAAAGACAgggtggtg	17.59	0.15	14.95	0.47	[67]
51	rs1402667001	g−34a	tggagg**a**ggACAAAGACAgggtggtg	17.74		15.42		
52	*ASCL3*	WT	tcgaaaaaTAAAA**T**AAAAtaaaacat	19.04	−0.29	18.24	−0.34	
53	rs1049743008	T−45C	tcgaaaaaTAAAA**C**AAAAtaaaacat	18.75		17.90		
54	*NOS1*	WT	tgtttcctGATA**G**AAAaaaaaaaatgg	18.56	0.34	18.77	0.18	
55	rs1195040887	G−27A	tgtttcctGATA**A**AAAaaaaaaaatgg	18.96		18.95		

Note. For each TBP–ODN complex, −ln(K_D) and Δln(K_D) = ln($K_{D;WT}$/$K_{D;min}$) are absolute and relative estimates (i.e., compared to those of the wild-type allele, WT), respectively, of the equilibrium dissociation constant expressed in natural-logarithm units (ln units). Human genes: *ABCA9*, ATP-binding cassette subfamily A member 9; *ASCL3*, Achaete-Scute family BHLH transcription factor 3; *ASMT*, acetylserotonin O-methyltransferase; *CDY2A,* chromodomain Y-linked 2A; *EPOR*, erythropoietin receptor; *F3* and *F9*, coagulation factors III and IX, respectively; *GCG*, glucagon; *GRIN1*, glutamate ionotropic receptor NMDA type subunit 1; *GTPBP6*, GTP-binding protein 6; *HBB*, *HBD*, and *HBZ*, hemoglobin subunits β, ծ, and ζ, respectively; *IL1B*, interleukin 1β; *LEP*, leptin; *MBL2*, mannose-binding lectin 2; *NOS1*, nitric oxide synthase 1; *SHOX*, short stature homeobox; *TPI1*, triosephosphate isomerase 1; *ZFY*, zinc finger protein Y-linked.

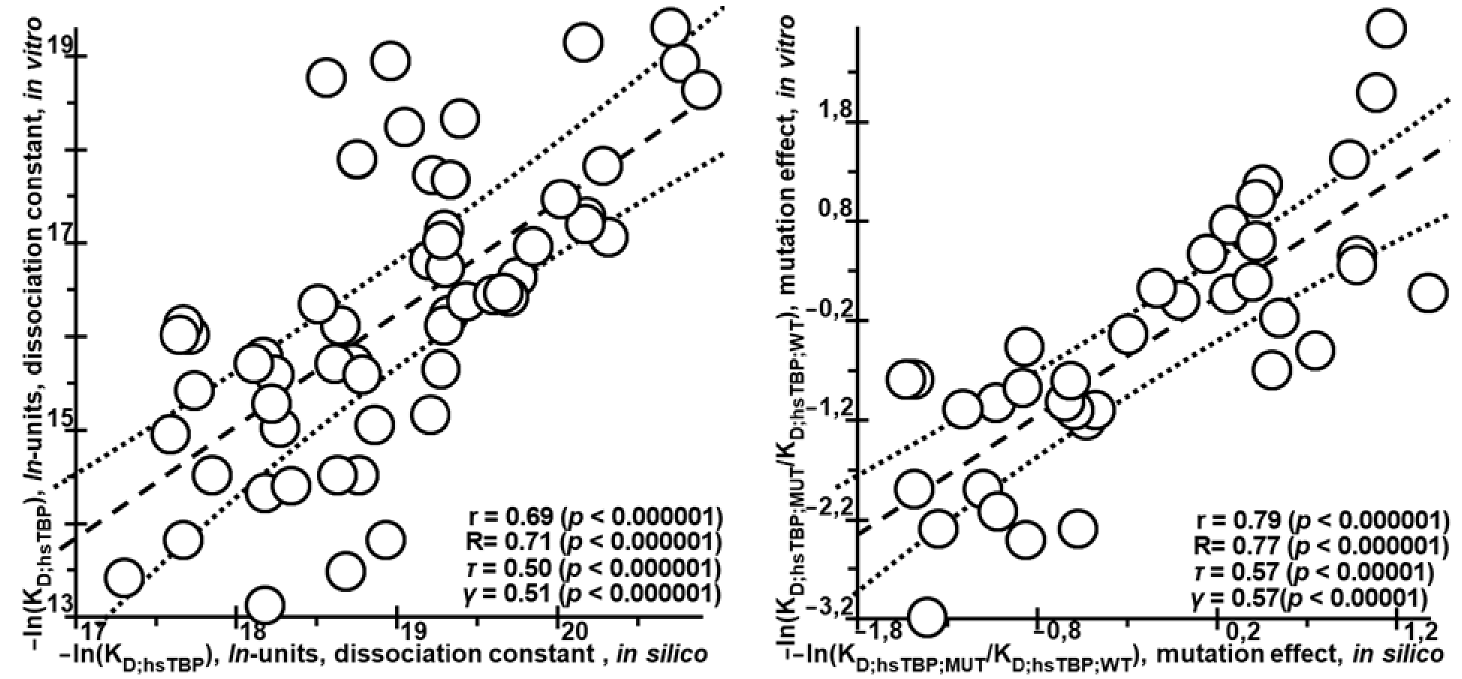

(a) (b)

Figure S1. The significant correlations between the *in silico*–predicted (X-axis) and *in vitro*–measured (Y-axis) K_D values of the equilibrium dissociation constant of the TBP–ODN complex, as graphical representation of Table S1. Legend: (a) and (b): absolute and relative estimates (i.e., compared to those of the wild-type allele, WT), respectively, of equilibrium dissociation constant K_D expressed in natural-logarithm units (ln units). Dashed and dotted lines denote linear regression and boundaries of its 95% confidence interval, as calculated in the Statistica software (Statsoft™, Tulsa, OK, USA). Circles denote the ancestral (WT) and minor alleles (dbSNP ID [66]) of the SNP listed in Table S1; r, R, τ, γ, and p are linear correlation, Spearman's rank correlation, Kendall's rank correlation, Goodman–Kruskal generalized correlation, and their statistical significance levels, respectively.

© 2022 by the authors. Submitted for possible open access publication under the terms and conditions of the Creative Commons Attribution (CC BY) license (http://creativecommons.org/licenses/by/4.0/).
